# Barriers and Benefits of the Scheduled Telephone Referral Model (DETELPROG): A Qualitative Approach

**DOI:** 10.3390/ijerph18105280

**Published:** 2021-05-16

**Authors:** Luis Miguel Azogil-López, Valle Coronado-Vázquez, Juan José Pérez-Lázaro, Juan Gómez-Salgado, Esther María Medrano-Sánchez

**Affiliations:** 1 Health Service of Andalusia, Primary Health Center Bollullos de la Mitación, 41110 Seville, Spain; luismiazogil@gmail.com; 2 Rural Medicine Group, Andalusian Society of Family and Community Medicine (SAMFyC), 18001 Granada, Spain; 3 Health Service of Castilla La Mancha, Primary Health Center Illescas, 45200 Toledo, Spain; mvcoronado@msn.com; 4 Department of Health Sciences, “Santa Teresa de Jesús” Catholic University of Avila, 05005 Ávila, Spain; 5 Andalusian School of Public Health, 18080 Granada, Spain; juanjose.perez.easp@juntadeandalucia.es; 6 Department of Sociology, Social Work and Public Health, Faculty of Labour Sciences, University of Huelva, 21007 Huelva, Spain; 7 Safety and Health Postgraduate Program, Universidad Espíritu Santo, Guayaquil 091650, Ecuador; 8 Faculty of Nursing, Physiotherapy and Podiatry, University of Sevilla, 41013 Sevilla, Spain

**Keywords:** qualitative research, referral, electronic consultation, telemedicine, primary care, waiting lists, patient safety, quality of healthcare, primary care physicians, hospital attending physicians

## Abstract

The recently developed scheduled mobile-telephone referral model (DETELPROG) has achieved especially important results in reducing waiting days for patients, but it has been decided to explore what barriers and positive aspects were detected by both primary care physicians (PCPs) and hospital attending physicians (HAPs) regarding its use. For this, a qualitative descriptive study was carried out through six semi-structured interviews and two focus groups in a sample of eleven PCPs and five HAPs. Interviews were carried out from September 2019 to February 2020. Data were analysed by creating the initial categories, recording the sessions, transcribing the information, by doing a comprehensive reading of the texts obtained, and analysing the contents. The results show that DETELPROG gives the PCP greater prominence as a patient’s health coordinator by improving their relationship and patient safety; it also improves the relationship between PCP and HAP, avoiding unnecessary face-to-face referrals and providing safety to the PCP when making decisions. The barriers for DETELPROG to be used by PCP were defensive medicine, patients’ skepticism in DETELPROG, healthcare burden, and inability to focus on the patient or interpret a sign, symptom, or diagnostic test. For HAP, the barriers were lack of confidence in the PCP and complexity of the patient. As a conclusion, DETELPROG referral model provides a lot of advantages and does not pose any new barrier to face-to-face referral or other non-face-to-face referral models, so it should be implemented in primary care.

## 1. Introduction

The DETELPROG (“Scheduled Telephone Referral”, from its acronym in Spanish) is a referral model that improves patients’ waiting times and unnecessary commuting with respect to face-to-face referral. It also considerably decreases waiting days for the resolution of the process through which the patient was referred to hospital, avoiding second face-to-face consultation with the hospital physician in almost all cases [1,2]. The evolution of this sophisticated and protocolised health attention model requires greater involvement and prominence of both primary care physicians (PCPs; *Médicos de Atención Primaria*—Spanish acronym MAP) and hospital attending physicians (HAP; *Médicos de Atención Hospitalaria* or *Internistas*—Spanish acronym MI), who should function to support primary care and not as a distinct level of healthcare [3]. Countries with prioritised primary care investments are better prepared to achieve sustainable development goals than those with hospital-centred systems [4]. This need has already been perceived by the Spanish government and the regional government of Andalusia, where our research project is developed, creating two strategic plans to strengthen primary care health attention within the public health system. In this coordination between rural or urban health centres and hospitals, the development of information and communication technologies is fundamental [5,6].

For the coordination between primary care and hospital care, there is strong evidence in favour of electronic referral interventions and interventions that include consultations with specialists prior to referral [7], which improve the problem of waiting lists for specialised consultations that are of so great concern in a large number of developed countries [8,9,10,11], and of course in Spain, where the waiting time average is 88 days [12], being this the main problem perceived by citizens regarding the health system [13]. The information and communication technologies implemented for this need can be classified into secure virtual asynchronous consultation platforms between primary care and hospital care (e-consultation), real-time mobile-phone consultations (curbside consultation), and scheduled mobile-phone or video conferencing consultations.

E-Consultations are platforms that have been widely developed in many countries [7,8,9,10,11,14,15,16,17,18,19,20,21,22,23,24,25,26,27] with a positive impact on accessibility, avoidance of unnecessary commuting and consultation, waiting times, training of professionals, communication and interprofessional information, acceptability, cost, and satisfaction of patients and professionals, although there are few data on morbidity and mortality [8,14,27].

As barriers, e-consultations require significant technological changes, investment, institutional involvement, leadership, and unpaid work burden for professionals. In addition, there are legal doubts about liability among professionals, occasional delays in receiving responses, and difficulties in monitoring patients and convincing managers and physicians to implement and use, respectively, e-consultation [21,22,26].

On the other hand, real-time mobile-phone consultations (curbside consultation) are a common practice in which benefits are obtained in waiting times, decreased face-to-face appointments in specialised consultation, and patient and professional satisfaction [3]; but also serious communication problems, incomplete or fragmented information, difficulty choosing the colleague to consult, unpredictable interruptions with unscheduled time expense, etc., usually related to the unschedulable aspect of such queries [28,29,30,31,32,33,34].

Finally, scheduled mobile-phone or videoconferencing consultations have generally been implemented in rural areas where patients have significant accessibility issues to hospital care consultations. In this sense, they are similar to traditional face-to-face consultations regarding clinical efficacy [35], and also share benefits, without sharing barriers, with the above consultation methods [1,2,36]. However, they do require expensive technology and there are still few studies on this matter, especially in the field of psychiatry.

The research team developed the DETELPROG, which achieved very important results in reducing waiting days for patients. However, on the other hand, it is surprising the low utilisation of this new referral model by PCP [1,2]. As a result, it has been decided to explore, through qualitative methodology, what barriers and positive aspects were detected by both PCP and HAP regarding the use of DETELPROG referral with the aim of finding areas of improvement and aspects to enhance in a possible, more widespread implementation in a rural health area or in other related areas.

## 2. Materials and Methods

### 2.1. Design

Qualitative descriptive study where a semantic content analysis was performed through six semi-structured interviews and two focus groups.

### 2.2. Study Population

The study population was a finite population made up of the five HAPs who provided consultation at the Rio Tinto hospital (the internal medicine service consists of seven HAPs in total) and the 13 PCP who formed the pilot group of the DETELPROG study [1,2], who had the possibility of receiving or performing scheduled mobile-phone referrals during the study period (there are 58 PCP with medical quota in the health area).

**Figure 1:**
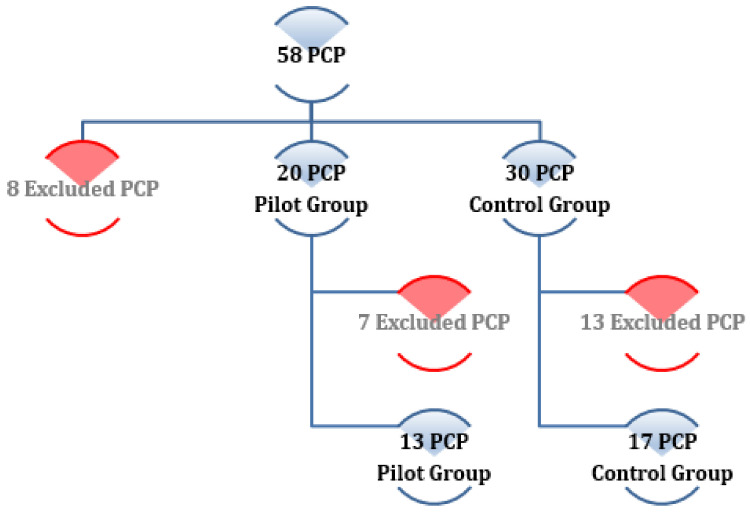
Primary Care Physicians’ sampling selection scheme of the DETELPROG study [1,2]. PCP: Primary Care Physicians.

The group of five HAPs was composed of two key informants, who were interviewed through a semi-structured interview, and three HAPs in the focal group. Of the 13 PCP, two of them were discarded from the study because they did not need to perform any referral to internal medicine in either period of the study, resulting in 11 PCPs (two key informants who were interviewed through a semi-structured interview, adding to the nine PCP in the focal group). Figure 1 shows the randomised selection process of PCP at the start of the DETELPROG project [1,2].

The sociodemographic variables of the sample are found in Table 1. There were three women and two men in the HAP group, aged between 45 and 67 years (median = 60; mean = 57.4; SD = 9.2). Two of the HAPs had worked as PCPs at the beginning of their working lives. As for the years of work experience, they had worked between 19 and 41 years, with a median of 37 years. With respect to PCPs, six of them were women and seven were men, aged between 37 and 66 years (median = 60; mean = 54.55; SD = 10.76).

### 2.3. Selection of Participants

- Inclusion criteria: Being a finite and accessible population, the study was carried out with all the members of the study population who wished to participate in it.
- Exclusion criteria: those PCP and HAP who did not participate in the DETELPROG study or any referral process.
- Key informants: They are participants who, due to the following characteristics, are interesting enough to have their opinions analysed through a semi-structured interview in a deeper way.
  o  Key informant PCP 1: the PCP that most rejected DETELPROG (21 rejections of 36 referrals).
  o  Key informant PCP 2: the PCP that least rejected DETELPRG (0 rejections of 14 referrals).
  o  Key informant HAP 1 and 2: two HAP who had worked in primary care as PCP years earlier were selected for their dual perspective.

### 2.4. Data Collection

A focus group was created for PCPs (nine participants) and another one for HAPs (three participants). Subsequently, four semi-structured interviews were done with the key informants, two with the PCP key informants, and two with the HAP key informants. Recordings were made from 3 September 2019, when the PCP focus group was created, to 28 February 2020.

The interviewer-moderator, in all cases, was the main researcher, workmate PCP of all the participants. An observer was also used for nonverbal language annotation for the focus groups.

Prior to the call and with the aim of not disregarding any barriers or benefits detected by the interviewees, researchers agreed to develop an interview script (see Files S1 and S2) asking about DETELPROG’s barriers and benefits as compared to face-to-face referral, at each stage of the interview (from the PCP’s referral to discharge by the HAP), and also taking into account the problems identified in studies on other non-face-to-face referral models found in the literature review.

### 2.5. Barriers in Data Collection

For the organisation of the PCP focus group, many difficulties arose for the call, but we managed to summon the participants in a restaurant located at equidistant distance from the work centres of the PCPs, and the recording was made at the beginning of the meal.

For the organisation of the HAP focus group, participants preferred to make the recording in the meeting room of their service without any convening problems. The last 5 min were not recorded because the memory of the recorder got full, and it stopped recording. To solve this setback and because both the interviewer and the observer agreed that there was no relevant information in those unrecorded minutes, (the problem was detected within few minutes of finishing the focus group interviews), it was decided not to incorporate anything else into the transcription of the recorded audio.

Individual interviews were conducted without any problems, by appointment, at the place and time proposed by the interviewee.

To perform semantic analyses, the main researcher had to be trained in the use of Nvivo version 12 software (QSR International, Melbourne, Australia) and then, train two other members of the group to make a correct triangulation when the time of analysis came. For the agreement, when establishing the final categories, the unanimous agreement was always reached for the designation of the category and for its definition. The agreement was also sought, at least by two of the three researchers, for the encoding of the data and of removed data by the three researchers whenever they considered these not useful.

### 2.6. Data Analysis

A content analysis of the collected data was carried out. Initially, the categories agreed by the research group and which emerged from the literature review were the following:

o  Decision making of the most appropriate type of referral.
o  Informed verbal and written consent.
o  Technical and programme characteristics for the request of the first appointment.
o  Communication with the HAP.
o  Technical details (phone, computer, …).
o  Interpersonal characteristics (decision making, attitudes, …).
o  Patient follow-up.
o  PCP-Patient relationship.
o  PCP-HAP relationship.

The subsequent process was carried out:

- Recording: interviews and focus groups were recorded in digital format with prior informed consent of the participants.
- Transcript: all recorded data were literally transcribed into a writting computerised processor.
- Comprehensive reading of texts: once the recordings were transcribed, a preliminary reading of these texts was made to correct transcription errors.
- Analysis of the contents: a semantic analysis was carried out in two phases:
  (1) Identification of relevant segments of the text (Encoding of transcriptions): Transcripts were encoded by 3 members of the research team, who subsequently agreed on the different categories and their final definitions (Table 2).
  (2) Profile analysis: Once the final categories were agreed and all recordings were transcribed, all transcripts were encoded and the transcription content analysis was performed by categories and divided by HAP and PCP profiles, with the help of the Nvivo 12 software (Table 3).

### 2.7. Ethical Considerations

The study was conducted according to the guidelines of the Declaration of Helsinki and approved by the Research Ethics Committee of the province of Huelva. In addition, for its randomised clinical trial phase, this project has been registered with clinical trial registration number ACTRN12617001536358.

Prior to the interview, the informed written consent of the participants was requested, and the protection and confidentiality of the information and personal data were guaranteed. The information obtained was treated in such a way that it was not possible to identify the participants. The processing of the data was done in accordance with Organic Law 15/1999 [37] on data protection and Royal Decree 994/1999 [38] on the security of automated files containing personal data.

## 3. Results

Most participants agreed that DETELPROG offers more prominence to the PCP as a coordinator of patients’ health problems, providing with more problem-solving capacity and allowing greater access to complementary tests, not currently available for PC, thus making the PCP part of the diagnostic and therapeutic decisions made by HAPs.

Comparing DETELPROG with immediate telephone means, PCPs and HAPs complained that immediate telephone means caused lower-quality work burden and information as it was not scheduled; only one PCP considered both means similar. Mailing was considered more impersonal and incomplete because it provided less information than DETELPROG and also offered more indeterminate times. The peace of mind it gives PCP to speak directly to their hospital workmate was greater than via email.

With regard to the overall assessment, for all PCPs, it was a very useful choice for referrals and, in all cases, they would use it on a regular basis if it were implemented. In the case of HAPs, this means was found useful, but with nuances (referral for easy matters, simple patients, request for a clearly indicated complementary test, for certain protocolised pathologies, among others).

On the ethical-legal implications, HAPs had doubts about the legal implications and their degree of legal liability in case of a problem appearing with any patient. In asking PCPs about this issue, they all felt that everyone had the responsibility for what they were doing.

In the preparation of the consultation for PCPs, which was a greater job than for a face-to-face referral, they did not express difficulties or points for improvement. No HAP had made any prior preparation.

All respondents agreed that DETELPROG improved the PCP-HAP relationship, but mostly in the long term, as in the case of this study, it involved few months and, thus, few mobile-phone referrals. As for the physician-patient relationship, there is a dichotomy between the parties: HAPs agreed that, with DETELPROG, the relationship was lost and, on the contrary, for PCPs, the relationship was strengthened.

PCP-HAP-patient communication was considered very positive by all interviewees, but while it is true that the quality and quantity of information was much richer when sharing the patient via DETELPROG than through the traditional paper format of face-to-face referral, the reliability of the information due to the fact that HAPs cannot physically see the patient raises concerns about the possibility of losing important patient data. When agreeing on the complementary tests, treatments, follow-up, reviews etc. to be carried out, there were different attitudes: following the proposals of the PCP, following those of the HAP, or conducting a constructive discussion between both physicians and the patient. In this regard, all the interviewees expressed enormous satisfaction in this regard, and there was always agreement between the parties, with the exception of a single occasion.

With regard to quantitative improvements in waiting times and decreased commuting, everyone was aware and highlighted them.

Regarding organisational characteristics, some participants complained about the lack of personal knowledge between PCPs and HAPs, that sometimes questioned the reliability of information and hindered understanding among physicians. The most permanent cited barrier was the schedule for phoning consultations, which were scheduled amid face-to-face consultations, and sometimes there were delays in HAPs responding to the PCPs because they were busy with an in-person patient in the consultation, something which also delayed PCPs and made HAPs uncomfortable for having their colleague waiting. The 15 min for DETELPROG were appropriate for the interviewees. It is also important to note that two HAP missed previous protocols to consider what type of patients to refer via DETELPROG and how to do so to make it faster and more operational.

For PCPs, it implied a work overburden, although it did not cause them a problem, as it was scheduled. For the HAPs, there was no overburden. There were no technical problems during DETELPROG when PCPs and HAPs were asked about them.

There was consensus that patients who were highly oriented by the PCPs and who clearly needed a specific test which was not accessible to PCPs clearly benefited from DETELPROG. However, as causes of rejection, the HAP focus group mentioned three scenarios: cases by not-well-oriented the PCP, mistrust in data exposed by the PCP, or because they did not find the PCP sufficiently trained to deal with the patient. In those cases, they requested an in-person consultation and started requesting some tests in advance. For one key HAP, there were no limitations except for the exclusion criteria from the study, and for the other one, this was not a suitable type of referral for complex patients, unless it was a patient already known to them. PCPs individually expressed that this means would not be indicated for patients who show a sign, symptom, or have a complementary test that they cannot interpret on their own or focus on it. Sometimes, defensive medicine is the cause of rejection. Also noted, as expressed by Key informant PCP 1, was the usual healthcare burden suffered by PCPs, which causes, in times of burden, to request a face-to-face referral, and which involves less workload. Table 4 shows the main topics where PCPs and HAPs coincide or differ, according to the study categories. Table 5 shows the proposals for improvement made by respondents for 6 of the barriers identified by themselves.

## 4. Discussion

DETELPROG has provided a reduction in patient commuting to hospital and an improvement in waiting times for a first consultation in internal medicine and for the resolution of the health problem for which the patient was referred [1,2]. In addition, it has been a very positive experience for both PCPs and HAPs and has given PCPs a more leading role as a manager of the healthcare of their patients [4], not only at the primary care level, but also in patients who need management from external hospital consultations. It has also provided PCPs with the ability to obtain complementary tests and treatments for their patients in an agile and consensual way with their hospital colleagues, for which they did not have independent access.

As fundamental advantages with respect to face-to-face referral, as well as e-consultation [21,22,26] and curbside consultation [3,39,40], DETELPROG has decreased waiting times, avoided unnecessary consultations, and improved the quality of face-to-face referrals and medical actions (diagnosis and treatment) by sharing the patient among physicians of both levels of care, thus improving the relationship between physicians, the PCP-patient relationship, and the satisfaction of all parties involved.

With regard to barriers, like e-consultation [21,22,26] and curbside consultation [3,33,40,41], the barriers that HAP noted the most were the loss of direct contact with the patients, which they cannot explore and have to rely on the information provided by the health centre physician, whom they often do not know. Nor are HAPs clear about the ethical-legal implications of their advice on a mobile-phone consultation where they do not physically see the patient, or the implications with respect to informed consent for some complementary tests whose requests they sign without verbally informing patients and having to delegate this essential activity to the PCPs, as is the case in the other models of non-in-person referral.

As compared with previous studies, DETELPROG avoids unnecessary face-to-face consultations, does not cause an overburden in physicians, does not require the implementation of new technologies, and improves specialised care response times, although response times are longer than with non-face models. Previous studies showed that the use of care protocols of healthcare phone calls on the part of receptionists, while trained to operate booking systems, is not effective for fully assessing and managing patients. Under these circumstances, face-to-face consultations looks more efficient than repeating a phone call. For this, immediate information offered by both physicians and nurses while the caller is still on the telephone can dramatically reduce the number of such contacts. In fact, the presence of a nurse acts as an effective filter under these conditions [42]. The same explanation can be offered for the decrease in hospital admissions observed during intervention periods. The literature contrasts with the present research when observing the experience from a triage telephone system that included general practitioners and primary care nurses. This study not only showed a decrease in work overload thanks to telephone consultations, but there were even more consultations, yet distributed among the health centre staff to find prompt and efficient assistance. So, the cost of the used resources did not vary with respect to regular consultations, but users did show their satisfaction for having been assisted on the same day of the consultation [43].

As it is a synchronous oral communication, the information transmitted in the three-way call between both physicians and the patient is of higher quality, more adapted to the needs of all the parties involved, more reliable, and richer, resulting in closer collaboration between physicians that makes the PCPs feel more supported when making diagnostic and therapeutic decisions.

Moreover, it does not require any extraordinary investment since all that was needed was a reorganisation of the services involved [21,22,26]. In addition, it avoided communication problems, incomplete or fragmented data, difficulty in choosing the professional colleague to consult, unpredictable interruptions with unscheduled time spent, etc., typical of immediate telephone consultations [28,29,30,31,32,33,34].

As for videoconferencing consultations, there is little data on their advantages and barriers beyond the field of psychiatry. Yet, it is possible to say that DETELPROG does not require technology for videoconferencing.

With respect to the Quadruple Goal to optimise health systems [44], it is considered to be a system that improves the health of the population by decreasing waiting times, which also improves diagnoses and treatments by being agreed by the three parties involved (PCP, HAP and patient), and optimises face-to-face referrals avoiding unnecessary ones. In this sense, there is an improvement in the cost of delays in waiting times and regarding the poor communication that exists in face-to-face referral between physicians (need to repeat complementary tests, repeat therapies, need for more complex therapies to have more evolved frames for longer waiting times, etc.), while, at the same time, lowering the costs for patients by not having to commute to the hospital. Likewise, it improves the satisfaction of medical professionals and, presumably, of the patients who receive the service, as has been manifested by the professionals themselves.

As limitations, DETELPROG is not considered to be a substitution, but a complementary model to face-to-face referral which can accelerate diagnosis and treatment but would not be indicated for patients who are not well oriented by PCPs, for patients who prefer a face-to-face referral, or for patients where the PCP has serious diagnostic doubts or lack of the necessary therapeutic resources. In this last case, DETELPROG may provide appropriate advice, while the in-person appointment, or an advance of such appointment, arrives. The usual care overburden of PCPs may be another limitation causing PCPs to opt, in high-pressure situations, for face-to-face referral in which they discharge responsibility for the study and follow-up of the patient in their hospital colleagues. Despite the high rejection rate by PCP (47%), PCPs express great satisfaction with DETELPROG and do not state important causes of rejection (defensive medicine, healthcare pressure, and the inability to orient the patient or interpret a sign, symptom, or complementary test).

The creation of two focal groups and six semi-structured interviews on a small sample (total of 16 professionals) could be regarded as limiting, but information was obtained from all the physicians who had participated in any DETELPROG and, though with several different nuances, the opinions were quite similar for each of both profiles so, though a bigger sample could add other data, we believe the sample size to be enough to obtain reliable results about the main barriers and benefits of the model.

As points for improvement, we believe that the organisation of regular meetings between physicians at both levels of care, as well as improving the time flexibility for mobile-phone consultation and having clearly stated, and in writing, the ethical-legal implications and a consensus document for monitoring patients with a predominant role of the PCP would be appropriate. It is considered appropriate to incorporate all these proposals, although in the case of implementing specific protocols we believe that it is an appropriate measure for all types of referrals and not specifically for DETELPROG.

## 5. Conclusions

DETELPROG is a referral model [1,2] that, besides reducing waiting days for patients and avoiding unnecessary commuting for hospital face-to-face consultations, with the associated benefits and, above all, in periods of pandemics such as the one we are facing now, implies a positive experience for those involved, giving PCPs a more prominent role. Also, it avoids the barriers associated to the lack of programmability of curbside consultations [28,29,30,31,32,33,34] and, as compared to e-consultations [21,22,26], improves the quality of the assistance received and of the information provided to HAPs, the relationship between primary care physicians and hospital attending physicians, and the costs are null. Thus, we consider it an ideal referral model to be used initially, leaving face-to-face referral for those cases agreed by both physicians via DETELPROG, which would imply an optimisation of the health resources, offering a more appropriate assistance to patients by avoiding unnecessary commuting to hospitals, with the associated risks, and without implying any cost.

## Figures and Tables

**Table 1:** Sociodemographic characteristics of sample.

Sociodemographic Characteristics	PCP (n = 11)	HAP (n = 5)
Sex n (%) Male Female	5 (45.45%) 6 (54.55%)	3 (60%) 2 (40%)
Age in years Minimum-Maximum Mean (CI 95%)	37–66 54.55 (47.31-61.78)	45–67 57.4 (46–68.8)
Work experience in years Minimum-Maximum Mean (CI 95%)	8–36 22.27 (15.97–28.58)	19–4132 (20.22–43.78)
Years in the same office at the beginning of the DETELPROG Minimum-Maximum Mean (CI 95%)	0–36 8.64 (1.34–15.93)	1–30 15.20 (1.73–28.67)
Distance to hospital in kilometres Minimum-Maximum Mean (CI 95%)	36 (±11)	-
Number of healthcare cards adjusted by age Minimum-Maximum Mean (CI 95%)	845–2679 1.756 (1330–2181)	-
Number of referrals in 2014 Minimum-Maximum Mean (CI 95%)	7–59 32.45 (20.62–44.29)	-
Rurality index Minimum-Maximum Mean (CI 95%)	−1.09/−0.1 −0.60 (−0.86/−0.32)	-

CI: confidence interval; HAP: hospital attending physician; PCP: primary care physician.

**Table 2:** Final categories.

Categories	Definitions
Primary Care Physicians as key axis	Feeling of giving the PCP a more key role in decision-making regarding the health of their patients, even in the hospital environment
Lack of available tests in primary care	As a cause of the DETELPROG’s success is the scarce tests catalogue available for PC, that allows expanding when agreed with the Hospital Attending Physicians.
Comparison with other types of referral	Comments and comparisons with other types of already experienced PC-hospital referral/contact (face-to-face referral, mail, non-scheduled telephone consultation…)
General qualitative assessment	General assessment and satisfaction of participants
Proposals for improvement	Proposals for improvements made by participants.
Ethical-legal implications	Doubts raised about ethical-legal issues
Causes for refusing-accepting DETELPROG	Patients and consultations characteristics, or any other type of characteristic that makes the PCP refuse DETELPROG or use it instead of face-to-face referral.
Positive or negative characteristics regarding:	
PCP-HAP relationship	Characteristics that modify the professional relationship between Primary Care Physicians and Hospital Attending Physicians
Physician-patient relationship	Characteristics affecting the PCP-patient relationship
Planning DETELPROG	Facilities or problems that have been raised since PCP considered referring the patient until the beginning of the telephone consultation (Planning suitability of the said referral method, informed consent of the patient, planning the day when the telephone consultation will take place, planning the telephone consultation with their workmate).
Quantitative improvements	Improvement of quantitative variables: waiting times, commuting, waiting lists, capacity to assist more/less patients.
Communication PCP-HAP-patient	Benefits and problems in telephone communication between PCP-HAP-patient (attitudes, agreement and disagreement on complementary tests, treatment, follow-up, revision, advice, quality of information, reliability of information, information record).
Organisational characteristics	Any characteristic that depends on DETELPROG organisation (initial training phase/prior preparation of participants, schedule organisation -time and duration-, work overburden, consultation protocolisation…).
Technical characteristics	Telephone performance, computer and software performance (access to patients’ medical history, complementary tests, consultation mode…)

HAP: hospital attending physician; PCP: primary care physician.

**Table 3:** Verbatim quote table per categories.

Category	Verbatim
1. Primary Care Physicians as key axis	“…the family physician increases his or her importance, his or her relevant role, increases decision-making; and, in the end, increases everything by, somehow, assuming more responsibility, though this was advised, agreed, etc., but the family physician becomes an even more relevant key axis in health care…” (key informant HAP 1)
2. Lack of available tests in primary care	“…Before HAP, I was a general physician here in Huelva and my training had dealt with hospital care right after finishing my degree, and I properly knew what to do. The thing is, I didn’t have the appropriate means. So, I suppose… that would be the issue, knowing what to do but having no means available… (key informant HAP 2) “…and then, something basic has been achieved, that is avoiding one step if we, primary care physicians, had the chance to request those complementary tests, more than 90% would be avoided...” (PCP focal group)
3. Comparison with other types of referral	
3.1 Immediate telephone call	“it was a somehow simpler consultation; I would say ‘I’ll request the CAT scan’, and then I would write: ‘The family physician is requesting a CAT scan, I find no contraindications so, I accept’, and everything was somewhat less informed because the family physician requested I prescribed a CAT scan, and the information I received was more or less sensible so I agreed but, well…” (HAP key informant 1)
3.2 E-consultation	“…it has been a long wait because one doesn’t know whether the person is available, maybe on holidays, or off-work or on leave… This happened to me, and then, I received the answer a month later. Also, you don’t feel so much at ease; the questions you may be asked by the specialist regarding the patient, maybe through an online question I write a series of data but there are still some missing that, in face-to-face communication, I would be able to better clarify…” (PCP)
4. General qualitative assessment	“My subjective feeling and that of my patients, which I can communicate to this group, is that it has been a highly positive experience, that is, the patient, as the introduction says, has had improvements in terms of time; he/she has been assisted earlier, has gained in comfortability, not only thanks to avoiding commuting to hospital, but also because the first consultation has been done in front of their family physician, who usually holds a much more intimate relationship with the patient than other specialists they don’t know”. (PCP focal group) “Very useful. I think this is already assumed; it is much more convenient and I would like this type of systems to be implemented. I feel more confident and at ease when contacting that person, having direct contact”. (PCP) “I serve as support, a good one that must be regarded as positive because this supportive element may save lives, but in certain circumstances, on the contrary, you may have overseen some issue and the patient would have benefited more from a face-to-face consultation despite this being a month and a half later in time; we must also be aware of this…” (HAP key informant 1)
5. Ethical-legal implications	“when you refer somebody to hospital, it is like you are getting rid of them, and then the hospital “internist” or specialist is in charge of their health or of that specific problem the patient has. However, this way, well, you can share the patient and the internist is not the sole responsible, but you are instead”. (PCP key informant 2) “…the responsibility is shared, and I’ll let you know why: from the very moment you start telling a hospital specialist about the patient, their history, medication and physical exploration, if that physician does not want to take care of the patient, he/she says, look, this patient…” (PCP focal group) “I must confess that, when this happened, I would say: ‘virtual consultation’, so that later on the fact that I didn’t physically see the patient would be known, that it was a telephone consultation and I had followed what my colleague from PC said, so it is true that I tended to say ‘better safe than sorry’, look, I did what I could by telephone…”. (HAP focal group) “so, obviously, there is a responsibility on the part of PCP, as I was saying, to accept these tests for their patient, but also, there is written responsibility that I requested the test so, if something goes wrong, if there is any negative effect, and the patient demands some explanation, this will go to the physician who requested the test, who hasn’t spoke to the patient, doesn’t know the patient…” (HAP key informant 1)
6. PCP- HAP relationship	“E3: It is not significant. The volume of consultations was not significant for that… E2: It was irregular”. (HAP focal group). “I think so, I think this strengthens the relationship, for example, physician Morales I didn’t know her, and she is so kind and collaborative in every way, and I believe this strengthens the relationship, sure”. (PCP)
7. Physician-patient relationship	“…when answering a telephone consultation, obviously, you cannot see the patient, that is completely out of sight to favour diagnostic speed, which is the actual interest, helping the patient as swiftly as possible…” (HAP key informant 1) “…it was like patients regarded you as something important, that is, as if in hospital you were more valuable and they agreed on opinions that had already been expressed to the patient and on which internists also agreed… What I experienced was an important improvement of the physician-patient relationship”. (PCP key informant 2)
8. Planning DETELPROG	“That is the only thing to change, some appointments, because they were finishing their on-call or they coincided with the specialist’s, which had been on-call as well. That is the only occasion in which we have had to change any appointment… In general, everything was properly done”. (PCP) “No, no. I believe I have a certain amount of experience at the clinical level and, overall, I am able to identify pathologies and solve the issue but, no, for instance, when you receive results and you have to analyse them and revise everything, yes…” (HAP key informant 2)
9. Quantitative improvements	“…one thing has mainly been gained, that is avoiding a step if we, primary care physicians, had the chance to request those complementary tests, this would avoid more than 90%...” (PCP focal group) “…everything was quite swiftly done…”. (PCP key informant 2)
10. PCP-HAP-patient communication	
10.1 Quality and quantity	“I believe that the family physician is quite enriching as when a patient is referred, you are lacking some data and the family physician does know the patient and, even sometimes, their family interrelationship, so there is a bunch of data that are not usually reported through written means but which, at the communication level, are even much more enriching, I would even say that this previous contact is quite enriching”. (HAP key informant 1). “…always, when talking to another person, be it even via phone call, communication is more fluent than what can be said in a written document, which is usually more objective information and, mainly because of time issues, also more limited”. (PCP key informant 1)
10.2 Reliability	“You are not told about the patient the same way depending on who gives you the report. In the end, you opt for requesting an abdominal ultrasound and requesting a consultation”. (HAP focal group). “…there is still a somehow uneasy feeling that I haven’t auscultated the patient, explored them, and I must only assume the data given by the PCP”. (HAP key informant 1)
10.3 Agreements	“E3: This is positive as the first days I request, and then, ‘how do we do it?’, ‘wait till I get informed’, ‘I think I kept the colleague’s phone number and Mari Ángeles, the assistant… (agreement on that). E2: But well, we did it like that, the first ones we referred them to the family physician with the signature and then, the last ones, we signed them here. If anybody wanted a test request or whatever, you would do so, if it was a special coding, and the test would be sent from here and the ultrasound examinations requests were done through the X-rays department and the appointment was programmed as if it had been done face-to-face”. E3: A record is sent, as if the patient were face-to-face, their record… E1: this is why you didn’t encounter any problem, right? E3: No… E2: Each one gave their best, right. (HAP focal group)
10.4 Attitudes	“…at the beginning there were some physicians or internists who were not willing to collaborate much, and we all know who we are referring to. So, this is an issue, lack of collaboration… for me myself and my circumstances…”. “Somehow sardonically they would say “virtual consultation” … At the beginning, internists were skeptical, that a secretary, a virtual model… It was not much heard at the moment but, little by little, they started taking it more seriously…”. (PCP focal group)
11. Organisational characteristics	
11.1 Preparation phase	“E3:…with the problem that you don’t know the interlocutor you are talking to, one gives it for granted that we, physicians, all have the same training… E1: that is questionable. E3: Well, that is why it also depends on who is talking to you in the interconsultation…” (HAP focal group)
11.2 Telephone consultation schedule	“Sometimes it occurred … that nobody answered the phone and the patient needed to go out of the office and wait for some minutes and, then, a quarter of an hour later while I was receiving other patients, we tried again and they answered… no appointment had to be canceled, it just took a bit longer… I received some more patients and, at the second try, it was solved”. (PCP key informant 1)
11.3 Time set for consultation	“As for the consultation times, it was 15 min; do you find it appropriate? “Yes, yes, absolutely”. (PCP key informant 1)
11.4 PCP work overburden	“maybe a bit of overburden for us who, instead of passing the patient over, we need to be more present and aware of what is happening in the medical history but, well, this is our job and what I like doing so, this overburden is not such…” (PCP key informant 2)
11.5 Prior protocols	“…it would be to, somehow, establish the assessment of, somehow, how to put everything together, all the available means and everything we can do to reach a consensus, that is, an appropriate method. Establishing protocols”. (HAP key informant 2)
12. Technical characteristics	“E1: Did you find any problem with telephone communication at the technological level, other from the one we already identified? E3: What I said before about changing room to answer the phone, but it was eventually solved… E2: no, no…”. (HAP focal group)
13.Causes for refusing-accepting DETELPROG	
13.1 Accepting	“This is for standard and easy issues, telephone consultation. For very specific minor issues.” (HAP focal group) “PCP-HAP communication, I believe there would be no a priori limitations. I think that updated and live communication where the family physician explains to the specialist the patient’s circumstance, a priori, the patient’s characteristics may not imply any limitation to reject the telephone consultation”. (HAP key informant 1) “I don’t think so, as telephone communication is simple and direct, and the patient may even be prevented from having to commute to hospital and, if the patient trusts the assisting physician, they will absolutely delegate to him/her”. (PCP key informant 1)
13.2 Refusing	
13.2.1 HAP	“You are not told about the patient in the same way, depending on the physician, and this is highly influential. You eventually end up requesting an abdominal ultrasound and setting an appointment”. “Older more complex patients or not so old but complex imply many nuances that are difficult to solve in a consultation, difficult to leave it solved, or I find it difficult unless both the PC physician and the specialist know the patient, that both know what the issue is about… that way, it could be possible”. “if I don’t know the patient’s issue, I don’t know how to explain if this is a bruise, or Velcro, or crackles. So, this must be seen by the internist… or if it is a skin condition whose origin I don’t identify and may be related to a more general disease…”. (PCP)
13.2.2 PCP	“If I don’t know the patient’s issue, I don’t know how to explain if this is a bruise, or Velcro, or crackles. So, this must be seen by the internist… or if it is a skin condition whose origin I don’t identify and may be related to any more general disease…”. (PCP) “In some odd case with a patient, being critical of myself, maybe due to my own feeling of responsibility, I think, I will make a mistake, not meet expectations…”. (PCP focal group) “Due to the shortage of time we’ve got, we don’t get rid of the patient because, the good point of referral is that you say ‘I’ll refer you to internal medicine, and they’ll take care of you, one less issue to care about, because I don’t have more time but, well… The downside would be that one, it is extra work added to what already is a quite complex schedule and agenda regarding times”. (PCP key informant 1)

HAP: hospital attending physician; PCP: primary care physician.

**Table 4:** Main points of agreement and disagreement between the PCPs and the HAPs related to barriers and benefits of a telephone referral model.

Benefits	PCP	HAP
Promotes PCP prominence as coordinator of their patients’ health problems	Repeated opinion	Repeated opinion
Improves availability of complementary tests for PCP	Repeated opinion	Repeated opinion
Avoids barriers for immediate telephone consultation	Repeated opinion	Repeated opinion
Works better than email consultations	Repeated opinion	Not expressed
General satisfaction	Very good	Good
Improves PCP-patient relationship	Repeated opinion	Not assessable
Improves PCP-HAP relationship	Repeated opinion	Repeated opinion
Improves information given to HAP	Repeated opinion	Repeated opinion
Perception of improvement regarding waiting days	Repeated opinion	Not expressed
Appropriate organisation for better DETELPROG	Repeated opinion	Repeated opinion
Adequate technical characteristics	Repeated opinion	Repeated opinion
Utility for a great majority of present-day referrals	Repeated opinion	Repeated opinion
BARRIERS		
No utility for certain patients	Distrustful patients, patients who don’t know how to focus, referrals when assistance is in high pressure moments	Patients wrongly directed by PCP, distrust in PCP, considering PCP not skilled to control the patient’s problem
Ethic-legal doubts	No doubts	Doubts
Worsens HAP-patient relationship	Not assessable	Repeated opinion
Certain insecurity of HAP for not meeting patients	Not assessable	Repeated opinion

HAP: hospital attending physician; PCP: primary care physician.

**Table 5:** Proposals for improvements for the detected barriers.

Barriers	Proposal for Improvement
When Primary Care Physicians called at the appointed time, Hospital Attending Physicians were busy with a face-to-face consultation	“I think the ideal would be to establish a fixed time to finish consultations and during which the physician would only be available in a relaxed atmosphere, concentrated and prepared in front of the computer, without carrying out any other activity, just being attentive to telephone consultations…”.
Issues regarding verbal explanation of the complementary tests consents	“the only way is for the family physician to have the requests in their office and, thus, be able to note down what has been consulted and agreed with the specialist, in some way”. (HAP key informant 1)
Responsibilities regarding patients’ follow-up	“I think the family physician needs to take up a preponderant role as they are in close contact with the patient… The specialist needs to review the tests... there would be clear issues, such as the patient having a neoplasm of the colon because, as in the case I was reporting before, a neoplasm of the colon… referring the patient to surgery or the corresponding specialist”. “…the close relationship between the family physician and the patient, well, maybe the simplest answer from the family physician would be ‘When you undergo the colonoscopy, come over here and let me know, and this way this close relationship would even favour a quick diagnosis”. (HAP key informant 1)
Lack of reliability in the information provided by Primary Care Physicians to Hospital Attending Physicians	“I think we should better know the physicians we count on in primary care before implementing something like this, so that we know who we are actually working with”. (HAP focal group)
	“maybe differentiating by sectors… perhaps the Valverde health centre, has such person as reference, or in Aracena health centre, so-and-so is the referred internist, that is what is sought today… Then, communication would improve as the 7 or 8 physicians of the area would know their reference internists…”. (HAP key informant 1)
Quality of information in telephone communication	“…it would be, somehow, establishing the assessment about how to put everything together in some way, all the available means and everything we can do to reach a consensus, that is, an appropriate method. Establishing protocols”. (HAP key informant 2)

HAP: hospital attending physician.

## Data Availability

All of this article is provided within the manuscript and its artwork. In addition, for its randomised clinical trial phase, this project has been registered with clinical trial registration number ACTRN12617001536358.

## Supplementary Materials

The following are available online at https://www.mdpi.com/article/10.3390/ijerph18105280/s1, File S1. Focus group script PCP-HAP; File S2. Interviews script PCP-HAP.
