# Supplementary material for: Barriers and Benefits of the Scheduled Telephone Referral Model (DETELPROG): A Qualitative Approach
Source: Int J Environ Res Public Health. 2021 May 16;18(10):5280. doi: 10.3390/ijerph18105280 (PMC8156098; doi:10.3390/ijerph18105280)
Supplement: Supplementary file 1 [file ijerph-18-05280-s001.zip › File S2. Interviews script PCP-HAP.pdf]

<u>**Presentation of the research team**</u>

Luis Miguel Azogil-López [1], Valle Coronado-Vázquez [2], Juan José Pérez-Lázaro [3], Juan Gómez-Salgado [4] and Esther María Medrano-Sánchez [5]

1. Valverde del Camino Health Centre (Huelva).
2. Directorate General of Healthcare. (Zaragoza).
3. Andalusian Public Health School (Granada).
4. University of Huelva.
5. University of Seville.

<u>**Introduction:**</u>

Waiting lists for specialised care consultation have become an international problem and reducing them is one of the challenges of all health systems in developed countries. As for the measures adopted and published by the different health systems at the international level, there are mainly two aspects: unscheduled immediate telephone consultations, and electronic consultations.

The first one is a telephone consultation made by the family physician to their hospital mate at the time they have a question during the consultation. Such consultations result in benefits in waiting times, decreased face-to-face appointments in specialised consultation, and patient and staff satisfaction, but there are also communication problems, incomplete or fragmented information, difficulty choosing the colleague to consult, unpredictable interruptions with unscheduled time expense, etc., usually related to the lack of programmability of this type of consultation.

Electronic consultations are secure chat or email platforms for family physicians to contact hospital colleagues for questions. This is the most internationally developed route with a general positive impact on measures of access, acceptability, cost, and satisfaction of patients and staff, although e-consultations require significant investments and institutional involvement, leadership, and working time for physicians.

As you may know, in our North of Huelva health area, the problems of waiting lists for consultations in specialised care are not alien to us, and we launched a clinical trial in 2015 with an innovative referral method consisting of a telephone consultation between primary care and internal medicine, but unlike the one mentioned above, in our case it was carried out on a scheduled basis, so we intended to avoid problems arising with immediate telephone consultation.

During the study, 72 scheduled telephone consultations were conducted in which a face-to-face consultation was avoided in 91% of cases (a more relevant outcome than in immediate telephone consultations (60%) and that in e-consultations [12%-85%]). In addition, it was possible to reduce waiting days for the first internal medicine consultation by 27 days, and the days until discharge by internal medicine by 46 with respect to patients in the control group who were referred in face-to-face consultations.

Although very positive quantitative results were obtained, we would like to know your opinion about your experience with this new referral method that has been created among all of us, with

the aim of finding strengths and possible improvements to optimize and be able to implement this model in our health area and export it to any other healthcare area that wants it. To do this, we will perform this work technique called a focus group in which we will ask several questions to the group and record your impressions to analyse them in detail and draw valid conclusions to improve our scheduled telephone referral.

Objectives of the study:

To detect barriers, facilitators, and elements of improvement to be able to implement scheduled telephone referral in our rural health area so that it can be replicated in other similar areas.

Presentation of the technique:

We propose to have an "open and spontaneous" dialogue about the scheduled telephone referral, so that each person can give their opinion. It is not necessary to strive to express in technical words; in order to understand (communicate) each one, you must express "in your own words". Like this meeting, several others will be held. The moderator, me myself, will guide the speech throughout the meeting to gather the greatest number of opinions from everyone.

"Don't be frightened by the recorder... the information you provide is "confidential". It is very important for us (the researchers) to have everything you say recorded "exactly as you say it, in your own words", that is why we ask for authorisation (important that it is done in writing) to record. The data collected is our work material. We couldn't take notes on everything you say "in general, we talk very fast and sometimes several people at once".

Presentation of the interviewee and the moderator:

Why have you been selected? A question that is always posed: Remember that all participants have been invited to participate because we believe that, as professionals participating in the DETELPROG study, you can provide highly relevant information on the topic we are interested in researching.

Resolving doubts before starting:

Do you have any questions before starting?

Primary Care Physicians individual interviews questions

1. What advantages and disadvantages have you generally seen in the scheduled telephone referral compared to traditional face-to-face referral?

2. What characteristics of the patient made you assess the **non**-adequacy of a scheduled telephone consultation for referral to Internal Medicine?

3. What difficulties or facilities did you encounter when explaining and proposing to your patients the scheduled telephone referral?

4. What barriers or facilitators did you encounter when requesting and scheduling the day of telephone referral?

5. What problems existed when establishing telephone communication with internal medicine and what advantages and disadvantages do you see with respect to a regular immediate telephone consultation?

6. What advantages and problems appeared during the telephone conversations established between the family physician, the internist, and the patient regarding the content of the information (quality of consultations and advice made and received), registration in digital history, duration of conversations, attitudes of those involved, access to patient information, over-work, ...?

7. What advantages, problems, and solutions were given for consensual telephone testing, treatments, advice, and controls?

8. What inconveniences and positive points did you encounter when monitoring the patient regarding the control of complementary test results, clinical evolution, patient responsibilities, need for further consultations or check-ups...)

9. Did you find any benefit or harm in the physician-patient relationship because of scheduled telephone referral?

10. And, with regard to the family physician-internist relationship?

11. State what you consider most beneficial and the biggest barrier you have encountered in scheduled telephone referral?

12. Do you think it would be useful to implement it in our health area and in any other area?

1. Useless

2. Little useful

3. Neither useful nor useless

4. Pretty useful

5. Very useful

Hospital Attending Physicians individual interviews questions

1. What advantages and disadvantages have you generally seen in the scheduled telephone referral compared to traditional face-to-face referral?

2. For which patient do you **NOT** consider a scheduled telephone consultation appropriate rather than face-to-face one?

3. What barriers or facilitators did you encounter when preparing the telephone consultation?

4. What problems existed when establishing telephone communication with the family physician and what advantages and disadvantages do you see with respect to a regular immediate telephone consultation?

5. What advantages and problems appeared during the telephone conversations established between the family physician, the internist and the patient regarding the content of the information (quality of consultations and advice made and received), registration in digital history, duration of conversations, attitudes of those involved, access to patient information, over-work, ...?

6. What advantages, problems, and solutions were given for consensual telephone testing, treatments, advice, and controls?

7. What disadvantages and positive points did you encounter when monitoring the patient regarding the control of complementary test results, clinical evolution, patient responsibilities, need for further consultations or check-ups...)

8. Did you find any benefit or harm in the physician-patient relationship because of scheduled telephone referral?

9. And, with regard to the family physician-internist relationship?

10. State what you consider most beneficial and the biggest barrier you have encountered in scheduled telephone referral?

11. Do you think it would be useful to implement it in our health area and in any other area?

1. Useless

2. Little useful

3. Neither useful nor useless

4. Pretty useful

5. Very useful
